# Zebrafish as a translational model for peptide-mediated synaptic dysfunction related to obesity

**DOI:** 10.3389/fnsyn.2026.1845252

**Published:** 2026-08-10

**Authors:** Amanda Costa Rodrigues, Adriel Parahyba Lacerda, Lincoln Takashi Hota Mukoyama, Gleyce Barrios de Carvalho, Júlia Aparecida Lima da Mata, Danieli Fernanda Buccini, Octavio Luiz Franco, Ludovico Migliolo

**Affiliations:** 1 S-Inova Biotech, Programa de Pós-Graduação em Biotecnologia, Universidade Católica Dom Bosco, Campo Grande, Brazil; 2 Centro de Análises Proteômicas e Bioquímicas, Universidade Católica de Brasília, Brasília, Brazil

**Keywords:** gut–brain axis, metabolic syndrome, neuroinflammation, obesity, synaptic plasticity, therapeutic peptides, zebrafish

## Abstract

Obesity-driven synaptic dysfunction is increasingly recognized as a key mechanism linking metabolic disorders to neurodegenerative diseases. This review aims to explore the utility of zebrafish (*Danio rerio*) as a translational model to investigate these mechanisms and screen peptide-based therapeutic strategies. Owing to their optical transparency, genetic tractability, and suitability for high-throughput screening, zebrafish provide a powerful platform for studying neuronal circuitry and evaluating bioactive peptides *in vivo*. Current evidence on pathophysiological processes underlying obesity-related synaptic impairment, including central resistance to metabolic hormones, oxidative stress, and the chronic inflammatory state, which promotes persistent immune activation and neuroinflammation. In addition, the persistence of these disturbances in the organism can lead to dysfunctions and pathologies, such as Parkinson’s and Alzheimer’s diseases, both of which are discussed in more detail in this review. Also, particular emphasis is placed on the roles of glial cells and the gut–brain axis in modulating synaptic integrity. We highlight how cutting-edge technologies, such as live neural imaging and single-cell transcriptomics, are accelerating peptide discovery and mechanism-of-action studies in zebrafish. Furthermore, we discuss emerging therapeutic peptides such as GLP-1 analogs and BDNF-based molecules that modulate inflammatory pathways, restore insulin signaling, and enhance neuronal resilience. Collectively, the evidence positions zebrafish as a robust model not only for elucidating disease mechanisms but also for advancing peptide-based interventions targeting synaptic dysfunction in obesity.

## 1 Introduction

Obesity is a metabolic disorder whose prevalence has been increasing exponentially, emerging as one of the major public health challenges of the twenty-first century (Tremmel et al., 2017). Fat accumulation is closely associated with the onset of other chronic diseases, such as cardiovascular disorders and endocrine conditions like type 2 diabetes mellitus (DM2), however, growing evidence points to an intrinsic relationship between obesity and a range of brain dysfunctions, including cognitive decline and the development of neurodegenerative diseases such as Alzheimer’s and Parkinson’s disease (Anstey et al., 2011; Piché et al., 2020). Despite the existence of other diabetic conditions, such as type 1 diabetes mellitus and gestational diabetes, DM2 accounts for 60 to 70% of cases and is strongly associated with the aging process (Szablewski, 2025). In this context, most studies investigating the interface between diabetes and dementia focus predominantly on DM2, particularly in the context of obesity, in which chronic low-grade inflammation, insulin resistance, and oxidative stress create a hostile cerebral environment that compromises synaptic integrity and function (Crispino et al., 2020).

The study of synaptic dynamics in response to systemic metabolic alterations is limited in traditional mammalian models due to high costs, long experimental durations, and difficulties in real-time visualization. In this context, the zebrafish (*Danio rerio*) emerges as a promising experimental model (Ghaddar and Diotel, 2022). Zebrafish combines conserved neuro-metabolic pathways with *in vivo* imaging capabilities and high-throughput screening, making it a powerful model for investigating obesity-associated neurodegenerative disorders (Santoro, 2014). These features position zebrafish as a valuable tool to investigate the interactions between obesity and synaptic health, as well as potential diagnostic strategies and novel therapeutic approaches (Phillips and Westerfield, 2014).

Given the advantages of zebrafish in therapeutic efficacy testing, its use becomes feasible for the identification of novel effective therapies, among which therapeutic peptides emerge as a promising class of drugs in neuroscience (Fernández et al., 2025). Peptides such as incretin analogs, exemplified by glucagon-like peptide-1 (GLP-1), and neuropeptides like brain-derived neurotrophic factor (BDNF) stand out for their high specificity and ability to act on multiple fronts of the pathophysiology of obesity and its neurological comorbidities (Cohen-Cory et al., 2010; Nauck et al., 2021). GLP-1 also exerts direct neuroprotective effects by modulating inflammation and oxidative stress, thereby supporting cerebral homeostasis (Lv et al., 2024).

In this context, the present review aims to examine the complex interplay between obesity and neurological dysfunctions, highlighting the study of underlying mechanisms and evaluating therapeutic interventions. Furthermore, the potential of therapeutic peptides to mitigate synaptic damage associated with obesity is discussed, emphasizing the value of this model as a strategic tool for developing diagnostic and therapeutic approaches to address the neurological consequences of this condition.

## 2 An integrative model: zebrafish in neuro-metabolic research

Neurological changes resulting from metabolic disorders have been extensively investigated using different animal models, including non-human primates, pigs, rodents, and, more recently, zebrafish, which has been gaining prominence in research aimed at understanding the effects of obesity (Kleinert et al., 2018). As an excellent tool in the field of neuroscience, zebrafish provide a better understanding of the mechanisms involved in plasticity, degeneration, and regeneration of brain tissue (Ghaddar and Diotel, 2022). Considered a translational model, in addition to a comprehensive analysis of the target disease, *D. rerio* also allows for the analysis of diagnoses and possible treatments (Phillips and Westerfield, 2014). To emphasize the relevance of zebrafish in global research, a bibliometric analysis using established databases identified 93,381 studies involving zebrafish, of which 20,291 were classified as relevant to toxicological research (Lima et al., 2026).

In fact, zebrafish is particularly well designed for large-scale genetic modifications due to its ex-uterine development, short generation time, and efficient transposon-based transgenesis systems (Moran et al., 2023). One of the main factors that motivates the use of zebrafish is their significant similarity to the human genome and its organization, especially in terms of the genetic pathways responsible for transduction (Postlethwait et al., 2000; Becker and Rinkwitz, 2012). In terms of physiology, zebrafish have the same metabolic organs as humans, reflecting a similarity in energy homeostasis, lipid storage, insulin secretion, and feeding-related behaviors (Zang et al., 2018). For this reason, when induced in *D. rerio*, obesity-related disorders are analogous to those observed in humans (Ghaddar and Diotel, 2022), driving the development of genetic models, with transgenic lines with obesogenic genes, or with obesity induced by high-fat diets (Zang et al., 2018).

**Figure 1:**
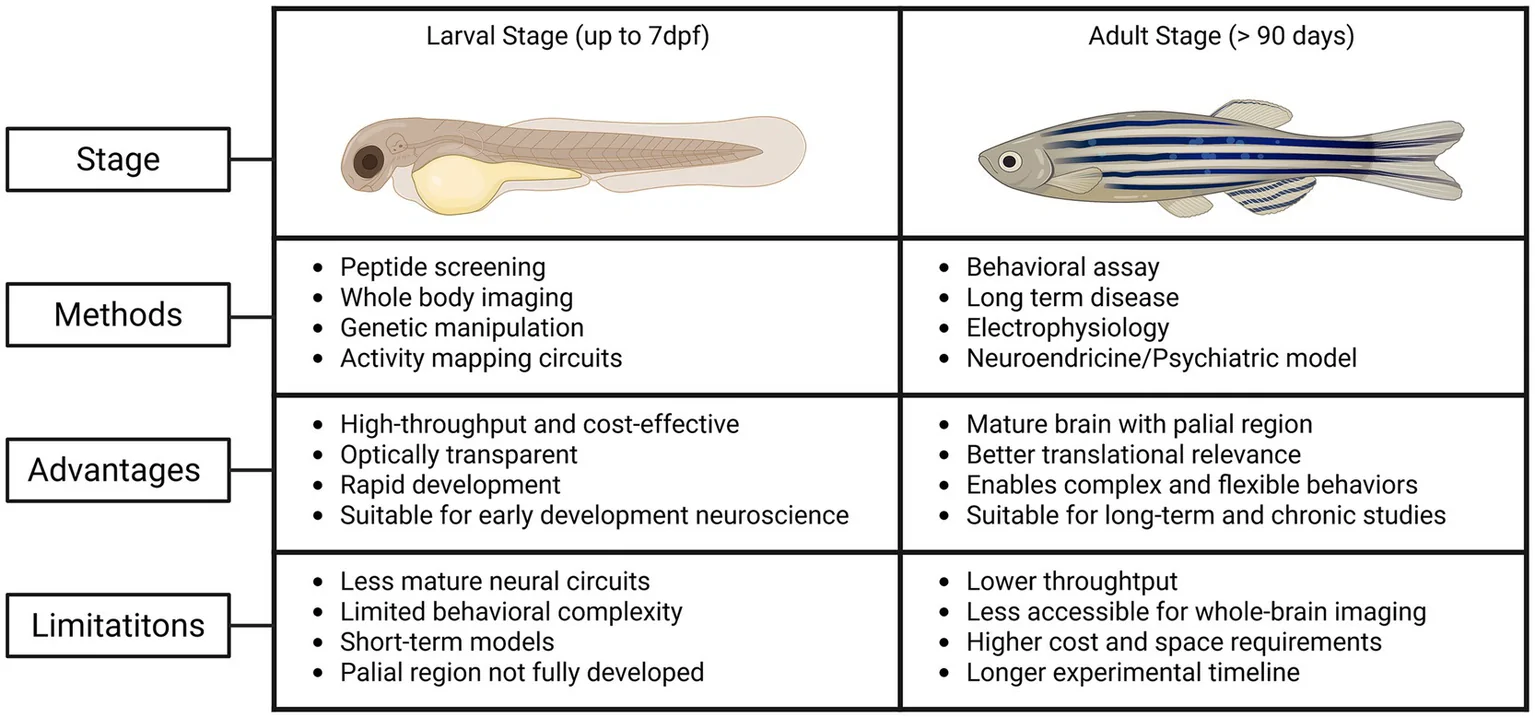
Experimental applications of zebrafish developmental stages in neuroscience research. Across development, from larvae (up to 7 dpf) to adults (>90 days), zebrafish enable the investigation of neural development, synaptic function, behavior, and disease-associated neurobiological alterations. Figure created and modified by *BioRender.com* .

Fluorescent reporter lines enable real-time visualization of developmental processes and obesity progression in transgenic zebrafish, supporting longitudinal *in vivo* studies (Driever and Fishman, 1996). Because of this peculiarity, fluorescent protein expression allows the monitoring of obesity in transgenic animals, from their development to progression (Faillaci et al., 2018). Despite their outstanding optical transparency, the neural circuits of zebrafish in the larval stages are still developing and do not yet function fully, as observed in adult fish (Shen et al., 2025). However, although they are not typically chosen for optical tests, adult fish possess anatomical regions similar in structure and function to the hippocampus (Zupanc et al., 2005) and amygdala of mammals (Perathoner et al., 2016), allowing for the introduction of behavioral tests in this model. Following this, we provide an example of the influence of the zebrafish’s developmental stages (larval and adult) on scientific research regarding neurological disorder (Figure 1).

Another relevant aspect is that, from a metabolic point of view, zebrafish express functional orthologs of genes involved in energy regulation, including Peroxisome Proliferator-Activated Receptors-*γ* (PPARγ), in addition to leptin and insulin receptors (Seth et al., 2013). Due to these factors intrinsic to zebrafish, this animal has also come to be used in the field of neuroscience, exploring the consequences of obesity and other metabolic disorders on brain homeostasis (Tonon and Grassi, 2023). These analogous disorders, such as genetic mutant lines like leptin receptor (*lepa* or *lepr*) knockouts are widely deployed to isolate endogenous neuroendocrine signaling pathways, consistently resulting in marked hyperphagia and automated adipose accumulation (Oka et al., 2010; Del Vecchio et al., 2021). Complementing these genetic frameworks, diet-induced obesity (DIO) paradigms utilizing high-fat diets or overfeeding with live prey (*Artemia salina*) successfully mimic the polygenic and environmental complexities of human metabolic syndrome, including systemic lipid shifts and severe hepatic steatosis (Oka et al., 2010; Fowler et al., 2021). Taken together, both modeling strategies establish a reliable dual-experimental baseline for investigating downstream neurobiological comorbidities, such as blood–brain barrier (BBB) disruption and localized synaptic dysfunction (Oka et al., 2010; Del Vecchio et al., 2021).

The observation of the translational boundaries of these models requires careful consideration of distinct technical and biological constraints. The ancestral genome duplication in teleost fish represents a major confounding factor, as targeted disruptions in specific genes like *lepa* can induce functional compensation by its paralog *lepb*, yielding divergent and context-dependent phenotypes across laboratory strains (Del Vecchio et al., 2021). Additionally, the regulatory feedback loops differ from mammalian systems, as zebrafish leptin is predominantly synthesized in the liver rather than acting as a classic adipocyte-derived signaling molecule (Michel et al., 2016; Del Vecchio et al., 2021). For dietary approaches, standardizing high-fat formulations remains challenging, and the associated hyper-alimentation often degrades tank water quality, introducing confounding environmental stress that shifts baseline cortisol levels (Smith et al., 2013; Michel et al., 2016). These challenges are continuously offset by the physical advantages of the model during imaging.

**Figure 2:**
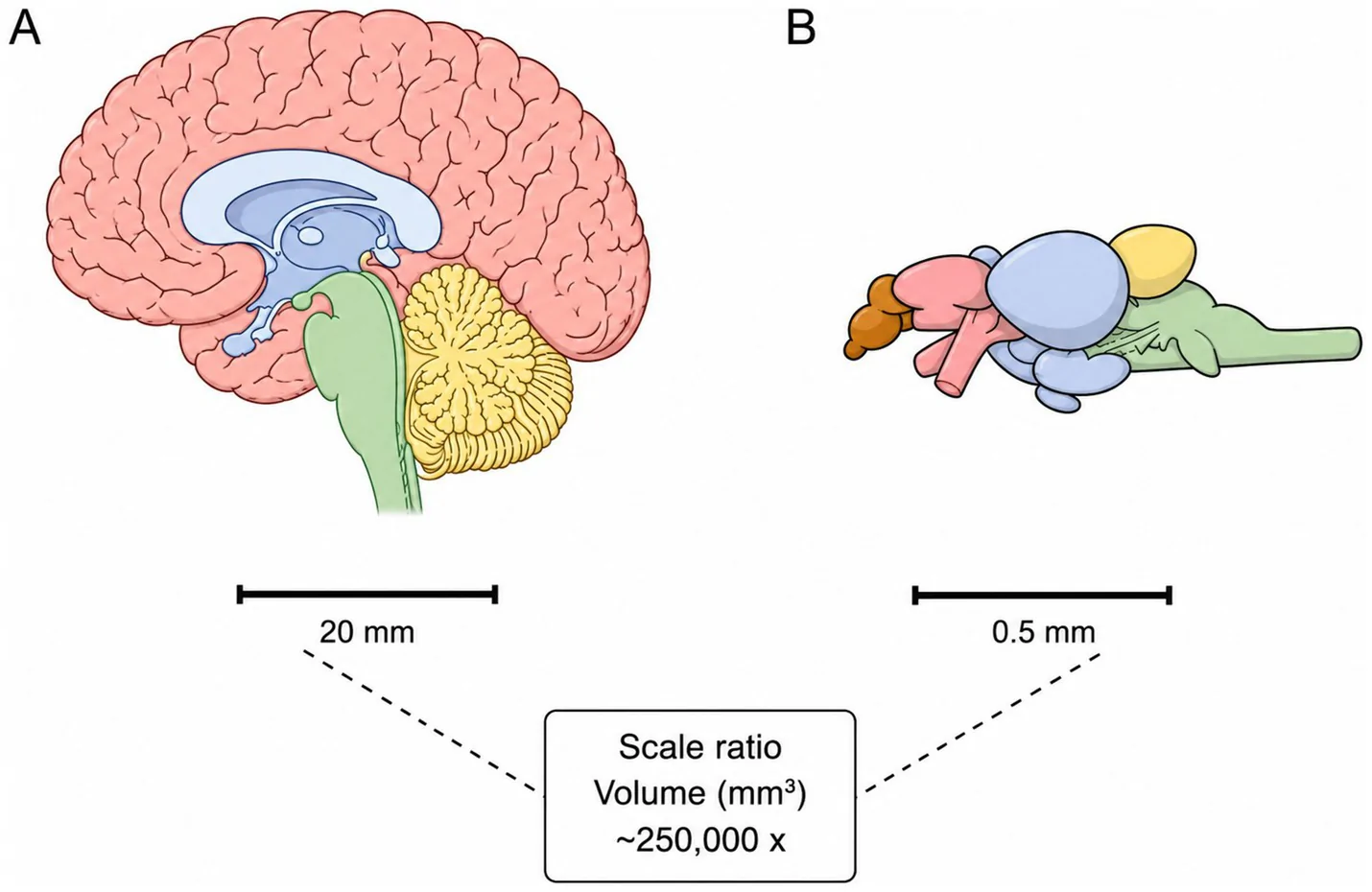
Comparative anatomy of the central nervous system in humans **(A)** and zebrafish **(B)**. The figure illustrates the distinct anatomical organization of homologous brain regions in both species. Brain structures are color-coded as follows: telencephalon (red), diencephalon (blue), brainstem (green), cerebellum (yellow), and olfactory bulb (brown, zebrafish only). Figure created and modified by *BioRender.com* .

In fact, a variety of studies warn about the relationship between increased body fat and the emergence of other chronic degenerative diseases, such as dementia (Hayden et al., 2006). In this context, other unique characteristics of zebrafish have emerged, such as the ability to regenerate the brain after major injuries without cell loss (Ghaddar et al., 2021), allowing for the exploration of neuroprotection and neurogenesis mechanisms (Dorsemans et al., 2017). Although the distribution of neurogenic niches in zebrafish is different when compared to humans (Grandel and Brand, 2013), generating a neuroanatomical distinction, studies point to homologous functions in several areas, such as those responsible for memory processes and fear responses (Fontana et al., 2018), making it an excellent model for studying diseases such as Alzheimer’s (Newman et al., 2014). Above, the image illustrates the comparative anatomy of the functional regions of the brains of an adult human and an adult zebrafish (Figure 2).

This animal model expresses glutamate and gamma-aminobutyric acid (GABA) receptors, with GABA acting as an inhibitory neurotransmitter in adults and as a neurotrophic factor during embryonic neuronal development (Kim et al., 2004). In this species, it is possible to observe in more detail the processes of synaptic plasticity, synaptic contact stabilization, and synaptic maturation due to the presence of Postsynaptic Density Protein 95 (PSD-95) (Meyer et al., 2005). Furthermore, susceptibility genes related to highly prevalent neurodegenerative diseases, including Amyloid Precursor Protein (APP) and presenilins (PSEN1/2), are conserved and functionally active (Joshi et al., 2009; Newman et al., 2009).

The preservation of endocrine and neurological pathways has turned zebrafish into an excellent integrated neuro-metabolic model, generating a better understanding of the pathophysiology of diseases correlated with these disorders (Yashaswini et al., 2025), also allowing for the possibility of new approaches to diagnosis and therapeutic treatments (Ochenkowska et al., 2022). One of the most unique contributions of the zebrafish model to translational neuroscience is the ability to perform dynamic and non-invasive imaging of biological processes in real time using an optical microscope (Ma et al., 2012). Among the various applications, imaging in studies such as the following can be highlighted: (i) Synapse dynamics: through complete visualization of the brain, it is possible to better understand the neural activity of cells and networks; (ii) Neuronal activity: through genetically modified calcium indicators, revealing subcellular changes in Ca^2+^ based on fluorescence intensity; (iii) Inflammatory response, cellular behavior during microglia recruitment; (iv) Cerebral blood flow and adequate oxygen transport to tissues (Schwerte et al., 2003; Leung et al., 2013; Hamilton et al., 2016).

Although rodents have been considered for a long time as the best animal model for testing new therapeutic approaches, zebrafish are gradually being introduced as a candidate for testing new drugs in the neuroscience field (Pound et al., 2004; Ibhazehiebo et al., 2020). In addition to being easy to maintain and low-cost, *D. rerio* can be used to perform *in vivo* toxicity and safety tests, reducing potential drug failures and allowing for faster evaluation of new compounds (Ochenkowska et al., 2022). As mentioned above, zebrafish are commonly used not only as a metabolic model, but also as a model that exhibits neurological comorbidities along with markers of neuroinflammation, oxidative stress, and reduced neurotrophic factors, establishing an experimental platform conducive to testing multifunctional therapies, such as peptides (Zhou et al., 2014; Shang et al., 2025). The versatility offered by this model is advantageous for the development of therapeutic peptides, since these bioactives require a better understanding of their pharmacokinetic and pharmacodynamic properties (Yang et al., 2016; Singh et al., 2024).

Recently, *in vivo* experiments have begun to use zebrafish in their studies to determine the efficacy and specificity of their therapeutic peptides (Shull et al., 2017). A study using zebrafish and peptide-based nanocomplexes proved that these bioactive compounds can be easily internalized in zebrafish embryos, do not cause harmful or toxic effects, and do not affect the normal development of the fish (Faria et al., 2024). Therefore, zebrafish transcend their role as a disease model to become an indispensable tool for therapeutic development, providing functiona*l in vivo* evidence acting on the obesity-synaptic dysfunction axis.

## 3 Obesity-induced synaptic dysfunction

Complex multifactorial metabolic condition that transcends the mere accumulation of adipose tissue to establish itself as a significant risk factor for the development of dysfunctions in the central nervous system (CNS) (Golkar et al., 2026). The deterioration of cognitive function, synaptic plasticity, and neuronal integrity represents one of the most concerning links between obesity and brain health (Crispino et al., 2020).

**Figure 3:**
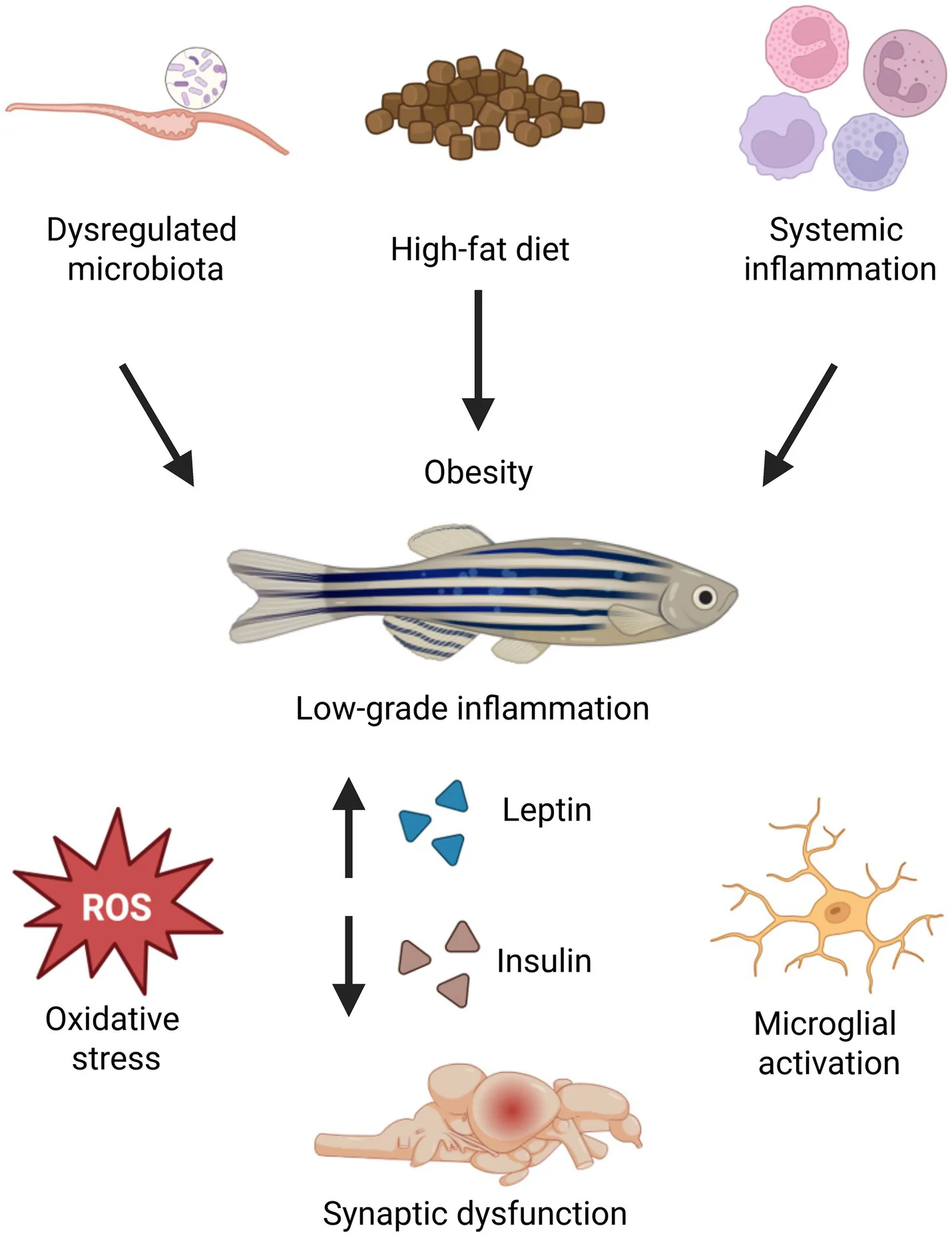
Zebrafish as a translational model linking obesity to synaptic dysfunction. The image illustrates how the consumption of a high-fat diet and microbiota dysregulation contribute to the development of obesity and systemic inflammation, leading to a state of chronic low-grade inflammation. This condition promotes increased production of reactive oxygen species (ROS) and oxidative stress, and disrupts leptin and insulin signaling, ultimately resulting in insulin resistance. Concurrently, microglial activation within the central nervous system exacerbates neuroinflammation, which may lead to synaptic dysfunction. Figure created and modified by *BioRender.com* .

Synaptic dysfunction represents a key pathogenic event that precedes both cognitive decline and neurodegeneration and can be marked by alterations in synaptic structure and function, impaired neuronal communication, and synaptic loss, in advanced stages (Valcarcel-Ares et al., 2019; Golkar et al., 2026). A deep understanding of the pathophysiological mechanisms underlying this dysfunction is essential for developing effective therapeutic and preventive strategies, particularly given that one of the major pillars of obesity-induced neurotoxicity lies in hormonal dysregulation, chronic inflammation (both systemic and neuroinflammation), and oxidative stress (Ghaddar and Diotel, 2022), as shown in the following schematic illustration (Figure 3).

Beyond hormonal dysregulation, disruption of redox homeostasis and activation of chronic inflammatory cascades are central events in obesity-associated neurotoxicity (Vuković et al., 2026). Among the mechanisms involved, the Nrf2 (nuclear factor erythroid 2-related factor 2) signaling pathway constitutes a major cellular defense against oxidative stress by regulating the expression of antioxidant enzymes (He et al., 2020). In zebrafish models of diet-induced obesity, excessive lipid intake has been associated with reduced Nrf2 expression in the telencephalon, accompanied by downregulation of key antioxidant genes, including superoxide dismutase 1 (*sod1*) and catalase (*cat*) (Weydert and Cullen, 2010; Meguro et al., 2019). Consequently, impaired antioxidant defenses favor reactive oxygen species (ROS) accumulation, which contributes to oxidative damage and compromises synaptic integrity (Beckhauser et al., 2016; Liu et al., 2025).

In parallel, obesity promotes activation of the Toll-like receptor 4 (TLR4)/nuclear factor kappa B (NF-κB) signaling axis. In zebrafish, activation of TLR4 by saturated fatty acids and systemic inflammatory mediators induces NF-κB nuclear translocation, leading to increased expression of pro-inflammatory cytokines such as interleukin-1β (IL-1β) and interleukin-6 (IL-6) (Campos-Bayardo et al., 2025). Sustained activation of these inflammatory pathways contributes to neuroinflammation and exacerbates synaptic dysfunction. Furthermore, stress-responsive mitogen-activated protein kinase (MAPK) pathways, particularly p38 MAPK and c-Jun N-terminal kinase (JNK), are activated in response to oxidative and inflammatory stimuli and have been implicated in neuronal damage and neurodegenerative conditions (Kim and Choi, 2015). Although these pathways have been extensively characterized in mammalian obesity models, their high degree of conservation in zebrafish highlights the utility of this model for investigating molecular mechanisms underlying obesity-induced neurodegeneration (Ghaddar and Diotel, 2022).

Starting with insulin and leptin, two crucial peptide hormones that regulate not only peripheral energy homeostasis but also brain function, acting as essential neuromodulators in the (Chang et al., 2019). Insulin crosses the BBB, and its receptors are abundantly expressed in brain regions involved in complex cognitive processes, such as the hippocampus and prefrontal cortex. In the brain, insulin signaling holds significant importance in modulating synaptic plasticity, directly influencing long-term potentiation (LTP) and long-term depression (LTD), which are fundamental cellular mechanisms for learning and memory formation (Chang et al., 2019; Crispino et al., 2020). Inflammation and oxidative stress contribute to increased BBB permeability by downregulating tight junction proteins in brain regions such as the hippocampus, thereby compromising barrier integrity (Davidson et al., 2014; Ghaddar et al., 2020). In mammalian models of diet-induced obesity, similar mechanisms have been associated with BBB disruption, neuroinflammation, and cognitive impairment, further supporting the translational relevance of these findings. Chronic metabolic inflammation associated with obesity is closely linked to activation of NF-κB signaling, which functions as a central regulator of pro-inflammatory cytokine expression and oxidative stress in the central nervous system. In rodent models of HFD-induced obesity, NF-κB activation in brain regions such as the hippocampus and hypothalamus has been associated with increased expression of pro-inflammatory mediators (TNF-*α*, IL-1β, and IL-6), leading to synaptic dysfunction, impaired neuroplasticity, and cognitive deficits (Davidson et al., 2014; Meguro et al., 2019).

In parallel, HFD exposure contributes to disruption of BBB integrity through downregulation of tight junction proteins, including claudin-5 and occludin, thereby increasing barrier permeability and facilitating peripheral inflammatory signaling into the brain. Comparable effects have been reported in zebrafish models of diet-induced obesity, in which increased oxidative stress, BBB leakage, and neuroinflammatory responses were observed (Ghaddar et al., 2020). Altogether, these data indicate that NF-κB-mediated inflammatory signaling represents a conserved mechanism linking metabolic dysfunction to BBB disruption and neurobehavioral impairment across vertebrate models, thereby supporting the translational relevance of zebrafish findings in the context of established mammalian evidence.

Similarly, leptin is an anorexigenic hormone produced by adipocytes which signals the state of energy reserves to the hypothalamus but also exerts direct effects on synaptic structure and function in several brain areas, including the hippocampus (Harvey et al., 2006). Leptin resistance, characterized by elevated hormone levels in the bloodstream without a corresponding cellular response in target tissues, impairs synaptic plasticity, neurogenesis, and contributes to cognitive and behavioral decline (Harvey et al., 2006; Ghaddar and Diotel, 2022). This failure in leptin signaling also promotes increased food intake and perpetuates the cycle of obesity and its neurological comorbidities.

Animal models, such as *D. rerio*, have proven valuable for investigating hormonal dysregulation and its effects on synaptic dysfunction. Studies in zebrafish subjected to high-fat diets (HFD) or overfeeding induce an obese state that mimics many aspects of the human condition, including insulin and leptin resistance (Godino-Gimeno et al., 2023; Al Jaberi et al., 2025). These models exhibit behavioral alterations, such as increased anxiety and deficits in memory and learning, which correlate with synaptic dysfunction and changes in the expression of genes related to plasticity (Picolo et al., 2021; Al Jaberi et al., 2025).

Also, obesity is intrinsically linked to low-grade chronic inflammation, which originates primarily from expanded and dysfunctional adipose tissue. This tissue, particularly visceral fat, releases a myriad of pro-inflammatory cytokines, such as tumor necrosis factor-alpha (TNF-*α*), interleukin-6 (IL-6), and interleukin-1 beta (IL-1β), in addition to chemokines and adipokines, into the systemic circulation (Salas-Venegas et al., 2022). The persistence of these inflammatory mediators compromises the integrity of the BBB, making it more permeable and allowing their translocation into the brain parenchyma. This invasion and the subsequent activation of glial cells, astrocytes, and microglia, initiate neuroinflammation, a pathological process that carries a substantial and deleterious role in synaptic dysfunction (Valcarcel-Ares et al., 2019; Salas-Venegas et al., 2022).

Chronic activation of microglia by peripheral inflammation or DAMPs drives their shift from a neuroprotective (M2) to a pro-inflammatory (M1) state, promoting the release of ROS and cytokines that impair neurogenesis, LTP, and synaptic integrity (Valcarcel-Ares et al., 2019; Crispino et al., 2020). Thus, the zebrafish model offers significant advantages for studying neuroinflammation and its impact on synaptic dysfunction, particularly because their larvae can be subjected to *in vivo* and real-time visualization of microglial activation, cell migration, and neuroimmune interactions using advanced imaging techniques (Ghaddar and Diotel, 2022). Also, the ability to trace individual neurons and synapses in larvae and adult fish enables detailed analysis of neuroinflammation effects on neural connectivity and function, providing valuable insights into the pathogenesis of obesity-induced synaptic dysfunction (Wanner and Vishwanathan, 2018).

The accumulation of ROS in the brain leads to oxidative damage to essential macromolecules, including lipids (lipid peroxidation), proteins (protein carbonylation), and DNA, thereby compromising neuronal structure and function and directly affecting synaptic integrity and communication (Crispino et al., 2020). In obesity, mitochondria are the main organelles responsible for cellular energy production (ATP) via oxidative phosphorylation, acting as a central pathological feature that manifests as reduced ATP production efficiency, increased ROS generation, and impaired mitochondrial dynamics and biogenesis (Crispino et al., 2020).

Recent studies have pointed to the dysregulation of specific pathways, such as that of cofilin-1, a protein that regulates actin cytoskeleton dynamics in dendritic spines, as a critical molecular link between obesity and synaptic loss (Alsegiani et al., 2025). Zebrafish, with its high metabolic rate and sensitivity to nutritional alterations, serves as an effective model for investigating oxidative stress and mitochondrial dysfunction in the brain. Obesity models in zebrafish demonstrate increased oxidative stress markers and changes in brain mitochondrial function, which correlate with observed cognitive and behavioral deficits (Picolo et al., 2021; Ghaddar and Diotel, 2022).

Indeed, *in vivo* models enable the investigation of obesity-induced synaptic dysfunction and its functional consequences, particularly through alterations in cognitive and behavioral performance. In zebrafish, these outcomes can be assessed using behavioral paradigms analogous to those employed in mammalian models, thereby facilitating translational comparisons. For example, adult zebrafish exposed to a long-term HFD exhibit significant impairments in learning, associative memory, and executive function, closely paralleling findings reported in rodent models (Meguro et al., 2019).

Beyond neuroinflammation, obesity-associated metabolic stress has also been linked to increased anxiety-like behaviors, which can be evaluated using the Novel Tank Diving Test (Picolo et al., 2021). These alterations are commonly characterized by increased thigmotaxis, heightened agitation, and reduced locomotor activity and exploration of the upper water column, collectively reflecting an anxiogenic phenotype (Borba et al., 2025). Additional behavioral paradigms, including the T- and Y-maze tests for spatial memory and the Novel Object Recognition test for recognition memory, provide complementary functional readouts for identifying cognitive deficits associated with obesity (Luo et al., 2024).

Taken together, these behavioral assays establish an important link between molecular alterations, such as obesity-associated synaptic dysfunction, and clinically relevant phenotypes, thereby strengthening the translational value of zebrafish models for investigating neurocognitive complications associated with obesity. Accordingly, Table 1 lists behavioral tests commonly employed in zebrafish and their mammalian counterparts.

**Table 1:** Translational behavioral approaches in zebrafish for the evaluation of synaptic dysfunction. The table summarizes behavioral tests used in zebrafish and their mammalian counterparts, highlighting the functional domains assessed, primary behavioral outcomes, and their relevance to synaptic function.

Functional domain	Zebrafish assay	Mammalian equivalent	Primary outcome and translational readout	Relevance to synaptic dysfunction
Anxiety-like behavior	Novel Tank Test	Open Field Test	Thigmotaxis and vertical exploration; reflects acute stress response (Johnson et al., 2023)	Linked to GABAergic/Glutamatergic imbalance in the pallium (Martins et al., 2024)
	Light/Dark Preference	Light–Dark Box	Scototaxis (dark preference); measures risk assessment and conflict (Johnson et al., 2023)	Reflects altered synaptic transmission in the ventral telencephalon (War et al., 2022)
Learning and memory	Y-Maze / T-Maze	Y-Maze / T-Maze	Spatial working memory and spontaneous alternation (Cleal et al., 2021)	Long-term memory formation depends on glutamatergic activation of plastic synapses (Blank et al., 2009)
	Conditioned Place Preference	Conditioned Place Preference	Associative learning and reward-seeking behavior (Yashina et al., 2019)	Dopamine receptor manipulations alter acquisition, consolidation, and probe performance in associative and latent learning tasks (Naderi et al., 2016)
Motor and sensorimotor	Visual Motor Response	Open Field / Activity Monitoring	Interaction between visual and motor centers (Bollmann, 2019)	These behaviors depend on distributed functional connectivity and circuit architecture, supporting their use as readouts of sensorimotor network integrity (Liu et al., 2025)
	Swimming Activity Analysis	Rotarod Test	Swimming speed, endurance-like output, and coordination patterns are common locomotor phenotypes in zebrafish behavioral analysis (Johnson et al., 2025)	Reflects motor neuron integrity and cerebellar-like synaptic coordination (Najac et al., 2023)
Social behavior	Shoaling Test	Three-Chamber Social Test	Shoaling quantifies social cohesion and group structure, including spacing and collective organization (Galstyan et al., 2022)	Oxytocin receptor loss increases group spacing and reduces polarization, linking shoaling phenotypes to oxytocinergic control of social circuit function (Gemmer et al., 2022)
	Mirror Test	Resident–Intruder Paradigm	Mirror-induced display assays quantify aggression and social reactivity in zebrafish (Pham et al., 2012)	Serotonergic signaling alters mirror-test behavior, supporting a link between aggression-like reactivity and serotonergic modulation of social circuit excitability (de Moura et al., 2023)

## 4 Dysfunction of non-neuronal cells in metabolic disorders

Astrocytes provide critical support in the energy metabolism of the CNS providing structural support and regulating the extracellular environment, such as ion balance and neurotransmitter removal, which are essential for synaptic function (Lopategui Cabezas et al., 2014; Chen et al., 2023). These cells modulate neuronal activity through the release of gliotransmitters, such as glutamate, adenosine triphosphate (ATP), and cytokines, and convert glucose into lactate, providing energy to neurons and supporting synaptic plasticity and memory (Bélanger et al., 2011; Beard et al., 2022; Chamaa et al., 2024). Metabolic disorders, such as obesity, can dysregulate this pathway, impair lipid supply, and affect synaptic function in the hippocampus (Suzuki et al., 2010). In this context, astrocytes are essential for lipid synthesis and cholesterol homeostasis, as they supply cholesterol to neurons through Apolipoprotein E (APOE), which is responsible for fat transport in the brain (Valenza et al., 2015).

Despite the physiological function of APOE in the brain, another gene expression, APOE4, alters the normal function of glial cells, potentially contributing to the risk of Alzheimer’s disease through protein aggregation and mitochondrial dysfunction (Fernandez et al., 2019). Overall, the presence of the APOE4 variant alters brain lipid metabolism, reducing the cholesterol available to neurons and leading to the loss of synaptic vesicles and a decrease in SNAP-25 protein, compromising the plasticity and functioning of the hippocampus *in vivo* (Jeong et al., 2019). According to studies, processes that affect lipid metabolism, such as those observed in metabolic disorders, contribute significantly to synaptic dysfunction and cognitive impairment (Yang et al., 2024).

Transgenic zebrafish models have been employed to study glial cells dynamically, allowing real-time observation of their development, regeneration, and response to nervous system injury (Lyons and Talbot, 2015). In a transgenic zebrafish model, the expression of the multifunctional protein DJ-1 in astrocytes protected against oxidative stress, being associated with the regulation of antioxidant and inflammatory proteins and the activation of the Nrf2-ARE transcriptional pathway, highlighting the crucial role of astrocytes in neuronal defense and preservation (Frøyset et al., 2018). Another transgenic model developed with green fluorescent protein (GFP) linked to glial fibrillary acidic protein (GFAP) enabled the visualization of astrocytes and other glial cells at different developmental stages and under pathological conditions (Bernardos and Raymond, 2006). These experimental models represent a valuable tool to investigate how metabolic alterations affect not only neurons but also other cell types, such as astrocytes, leading to secondary neuronal dysfunction and synaptic impairments (Chen et al., 2020).

Among the various cell types affected by metabolic alterations, microglia stand out for their immunological role and their close relationship between metabolism and neuroinflammation (Wake et al., 2011). Microglia not only detect and respond to pathogens or injuries but also regulate the synaptic microenvironment by controlling the availability of metabolites and influencing neurotransmitter uptake and recycling, thereby contributing to synaptic remodeling (Crapser et al., 2021). In this way, microglia maintain a balance between neuronal activity and inflammatory signaling, being essential for the preservation of synaptic plasticity (Deczkowska et al., 2018). Studies have shown HFD induce microglial activation in the hypothalamus, a critical region for the regulation of food intake and energy expenditure through the TLR4/NF-κB pathway, leading to the release of pro-inflammatory cytokines such as TNF-*α* and IL-1β, which are closely associated with the onset of neurodegenerative diseases (Stathori et al., 2025).

Another indicator of microglial activation is ghrelin (Ghre) signaling, a 28-amino-acid peptide that directly influences microglial activity in the context of obesity and type 2 diabetes mellitus (Pereira et al., 2017). Ghrelin modulates inflammatory and neurometabolic responses during states of peripheral insulin resistance and hyperphagia, leading to the presence of reactive microglial cells with reduced homeostatic efficiency, thereby promoting neuroinflammation and synaptic dysfunction (Kojima and Kangawa, 2005). Thus, dysregulation of the ghrelin–microglia axis links obesity, insulin resistance, and metabolic disturbances to cognitive impairment and the progression of neurodegenerative diseases (Russo et al., 2022). Excessive microglial activation is associated with the release of pro-inflammatory cytokines, which can induce synaptotoxicity and disrupt neural circuits involved in learning and memory (Wellen and Hotamisligil, 2005).

In adult zebrafish subjected to overfeeding, microglial activation is accompanied by increased levels of pro-inflammatory cytokines, oxidative stress, and reduced neurogenesis, with specific microglial subpopulations either modulating synaptic activity or phagocytosing apoptotic neurons, indicating the presence of distinct molecular programs that regulate adaptive and inflammatory responses in the brain (Ghaddar and Diotel, 2022). Consequently, obesity in zebrafish has been shown to impair short-term memory, while long-term memory remains relatively preserved (Godino-Gimeno et al., 2023). Moreover, metabolic disorders commonly associated with obesity, such as hyperglycemia and overnutrition, trigger a neuroinflammatory state characterized by intense glial activation, compromising the integrity of the blood–brain barrier and impairing neurogenesis, suggesting that the metabolic impacts on the brain are highly conserved across species (Ghaddar and Diotel, 2022). Despite these detrimental effects, the brain also activates endogenous regenerative mechanisms, implying that compensatory responses attempt to repair the damage induced by high-fat diets (Azbazdar et al., 2023; Sadeghdoust et al., 2024).

Oligodendrocytes also stand out as glial cells highly sensitive to metabolic alterations, and their dysfunction, often associated with myelin loss under neurodegenerative conditions, may precede and contribute to neuronal degeneration (Griffiths et al., 1998). Metabolic disturbances in oligodendrocytes, including reduced glycolysis, insufficient lactate transport, and impaired lipid synthesis, particularly cholesterol, compromise energy supply to axons and the maintenance of myelin, thereby impairing efficient neuronal conduction (Saher et al., 2005). In obesity, these effects are exacerbated by decreased cerebral blood flow and reduced oxygen and nutrient availability, which enhance oxidative stress and render oligodendrocytes even more vulnerable (Langley et al., 2020). Such processes lead to diminished mitochondrial function, impaired differentiation, and activation of apoptotic markers, hindering remyelination, aggravating neuroaxonal deficits, and contributing to long-term neurodegeneration (Neto et al., 2023). Overall, microglia, astrocytes, and oligodendrocytes can shift from an oxidative to a more glycolytic metabolic profile, promoting cytokine production, oxidative stress, and neuronal impairment; these metabolic alterations directly disrupt neuron–glia communication and drive the progression of neurodegenerative diseases (Afridi et al., 2020).

Therefore, these glial cells undergo a Warburg-like metabolic reprogramming characterized by an increased dependence on aerobic glycolysis and reduced mitochondrial oxidative metabolism (Vizuete et al., 2024). In the context of obesity, elevated levels of circulating saturated fatty acids and systemic inflammation act as key drivers of this metabolic shift, promoting glial dysfunction in the central nervous system (Guo et al., 2026). This process is mediated by the upregulation of hypoxia-inducible factor 1-alpha (HIF-1α) and glucose transporter 1 (GLUT1), leading to increased glycolytic flux and intracellular accumulation of lactate and reactive oxygen species (ROS) (Corcoran and O’Neill, 2016). These metabolic byproducts function as signaling molecules that promote activation of the NLRP3 inflammasome complex (Ralston et al., 2017). Upon assembly, the NLRP3 inflammasome activates caspase-1, resulting in the maturation and secretion of IL-1β and IL-18, which are central mediators of obesity-associated neuroinflammation.

In zebrafish models of diet-induced obesity, similar inflammatory and metabolic signatures have been reported, including increased oxidative stress and activation of conserved inflammatory pathways, supporting the translational relevance of these mechanisms across vertebrates (Wang et al., 2025). Importantly, this glial metabolic dysregulation contributes to impairment of the astrocyte–neuron lactate shuttle (ANLS), reducing metabolic support to neurons and leading to ATP depletion and structural instability (García-Domínguez, 2026). At the synaptic level, these alterations are associated with disrupted synaptic homeostasis, impaired neurotransmission, and synaptotoxicity (Hasan et al., 2026). In this context, peptide-based interventions emerge as promising modulators of obesity-induced neuroinflammatory and metabolic dysfunction. Bioinspired and rationally designed peptides have been reported to modulate key upstream regulators of this cascade, including HIF-1α signaling, GLUT1-mediated metabolic flux, and NLRP3 inflammasome activation, thereby attenuating IL-1β/IL-18 release and restoring glial metabolic balance (Wu et al., 2025, 2026). In addition, certain neuroactive peptides may directly influence synaptic stability by modulating neurotransmitter systems and promoting synaptic plasticity. Collectively, these peptide-mediated effects suggest a potential strategy to counteract obesity-driven glial dysfunction and synaptic impairment, with zebrafish providing a valuable translational platform for screening and functional validation of these compounds (Muraleedharan et al., 2020; Sun et al., 2020).

In this context, therapeutic strategies aimed at modulating neuroinflammation have garnered significant interest, with anti-inflammatory peptides emerging as promising candidates due to their ability to regulate the release of inflammatory mediators, modulate pattern recognition receptors, and inhibit pro-inflammatory signaling pathways (Liu et al., 2024). Moreover, zebrafish represent a valuable model for investigating the role of intracellular peptides in cell–cell communication, enabling studies on mechanisms underlying neurological disorders and neurodegenerative diseases (Teixeira et al., 2019). Evidence suggests that certain bioactive peptides can modulate the release of inflammatory mediators, regulate the expression of pattern recognition receptors, inhibit pro-inflammatory signaling pathways, and promote the resolution of inflammation, thereby restoring homeostatic balance in the central nervous system (Giri and Chandra, 2025).

## 5 Microbiota–gut–brain–synapse axis: insights from zebrafish models

The gut microbiota is increasingly recognized as a critical regulator of host metabolism, immune homeostasis, and bidirectional communication with the central nervous system through the microbiota–gut–brain axis (Weiss and Hennet, 2017; Moszak et al., 2020). Alterations in microbial composition associated with obesity promote intestinal dysbiosis, systemic inflammation, and metabolic dysfunction, ultimately contributing to neuroinflammation and synaptic impairment. Under normal conditions, the gut microbiota maintains host homeostasis and mitigates adverse physiological effects. However, factors such as dietary habits, medication use, alterations in the intestinal mucosa, immune dysregulation, and changes in microbial composition can disrupt this balance, promoting dysbiosis and metabolic disorders (Weiss and Hennet, 2017; Moszak et al., 2020).

In fact, individuals with reduced gut microbial diversity exhibit higher body mass index, adiposity, dyslipidemia, brain insulin resistance, and a more pronounced inflammatory state (Liu et al., 2017). Alterations in bacterial metabolism influence host biochemical diversity and metabolic activity, thereby affecting multiple physiological processes. Consequently, obesity-associated dysbiosis contributes to excessive adipose tissue accumulation and the exacerbation of inflammatory responses (Smith et al., 2007; Gomes et al., 2018).

Over the past decade, gut dysbiosis has been associated with numerous disorders, including obesity, diabetes, cardiovascular disease, autoimmune diseases, and neurological disorders (Martin-Gallausiaux et al., 2021). Increasing evidence indicates that specific microorganisms, their metabolites, and structural components modulate the pathophysiology of neurodegenerative diseases such as Alzheimer’s and Parkinson’s disease (Vogt et al., 2017). Although the precise mechanisms underlying these interactions remain incompletely understood, increasing evidence supports a central role for the microbiota–gut–brain axis in obesity-associated neurodegeneration (Zang et al., 2018; Zhong et al., 2022). Through *in vivo* studies, communication within the microbiota–gut–brain axis has been shown to occur through a complex bidirectional network in which the central nervous system regulates the enteric nervous system, autonomic nervous system, and microbial activity, while signals originating from the gut and its microbiota influence CNS development and function (Lynch and Pedersen, 2016; Socała et al., 2021).

One of the principal communication pathways within the microbiota–gut–brain axis is the vagus nerve, which transmits intestinal signals to the central nervous system (Bonaz et al., 2018; Ahmed et al., 2022). Microbial metabolites constitute another major route of gut–brain communication. These metabolites are end products of bacterial metabolism and profoundly influence brain function through interactions with the autonomic nervous system and synaptic signaling (Cryan et al., 2019; Kasarello et al., 2023; Miri et al., 2023). Among them, short-chain fatty acids stimulate the secretion of insulin, ghrelin, leptin, and amylin, thereby slowing gastric emptying, prolonging intestinal transit, and enhancing glucose-dependent insulin release (Koh et al., 2016; Martin-Gallausiaux et al., 2021).

Furthermore, the gut microbiota produces neurotransmitters and neuromodulators, including catecholamines, GABA, and tryptophan-derived metabolites, which influence hypothalamic activity, neuroendocrine signaling, and neurodevelopment (Cussotto et al., 2018; Wang et al., 2023). Another important communication route is the neuroendocrine pathway, in which enteroendocrine cells release hormones and neurotransmitters, including GLP-1, PYY, cholecystokinin, substance P, and serotonin, playing an essential role in communication between the intestine and the central nervous system (5-HT) (Wang et al., 2023). Therefore, tryptophan, metabolized by the microbiota into serotonin and kynurenine pathway compounds, may, under inflammatory conditions, be redirected to the production of quinolinic acid, a neurotoxic metabolite associated with neuronal death in neurodegenerative diseases (O’Mahony et al., 2015; Agus et al., 2018). Thus, studies with gnotobiotic zebrafish have been essential for mapping how the presence or absence of specific microorganisms alters the metabolite profile and, consequently, neuronal development and function (Rea et al., 2022).

An increasing number of studies have examined the bidirectional signaling within the brain–gut–microbiome (BGM) axis in the pathophysiology of obesity, mediated by metabolic, endocrine, neural, and immune mechanisms (Osadchiy et al., 2019). In this context, although structural differences exist between the zebrafish and human intestines, *D. rerio* shares conserved molecular mechanisms related to intestinal structure and function (Lieschke and Currie, 2007). Accordingly, several obesity models have been developed in zebrafish through dietary and genetic manipulations (Zang et al., 2018), enabling the comparison of metabolic phenotypes in DIO, such as overfeeding with a normal-fat diet (Landgraf et al., 2017).

Recently, several studies, many of which employ zebrafish, have elucidated molecular mechanisms by which the gut microbiota influences neurodegeneration (Wang et al., 2022). Molecular mimicry involves bacterial amyloids, such as the Curli protein from *Escherichia coli*, which are structurally analogous to human Aβ and *α*-synuclein and can trigger exaggerated immune responses, accelerating protein aggregation linked to neurodegeneration (Friedland, 2015). During dysbiosis, lipopolysaccharide from Gram-negative bacteria can cross the intestinal and blood–brain barriers, activating glial Toll-like receptors and promoting neuroinflammation via TNF-α and IL-1β, thereby contributing to neuronal damage in Alzheimer’s and Parkinson’s diseases (Sampson et al., 2016; Adedara et al., 2024). Zebrafish models are particularly advantageous for visualizing these processes in real time, enabling the observation of immune cell infiltration into the brain and microglial activation following systemic lipopolysaccharide exposure (Mohanta et al., 2020).

Given the complexity of these microbiota–gut–brain interactions, robust experimental models are required to investigate these pathways under controlled conditions. The zebrafish is a model organism known for its use in the field of translational neuroscience and has also been used in studies addressing the gut microbiome (Gerlai, 2014; Borrelli et al., 2016; Lu et al., 2021). The zebrafish provides insights into the causative role of certain microorganisms in neuroinflammation, BBB dysfunction, and the aggregation of pathological proteins such as beta-amyloid (Aβ) and alpha-synuclein (α-syn) (Lee et al., 2021; Zhang et al., 2022). Nevertheless, their capacity to create gnotobiotic models revolutionizes their use in studying the gut-brain axis, and, unlike mice, breeding germ-free zebrafish requires a rapid and low-cost process, achieved through surface sterilization of the eggs (Xia et al., 2022). Based on this approach, the correlation between the direct function of microbial components and brain health can be investigated, an advancement that was difficult to achieve with more complex models (Stagaman et al., 2024).

## 6 Technologies applied to zebrafish

The zebrafish model allows biological processes to be observed in real time, offering significant advantages over *in vitro* models or other opaque animal models, enabling imaging at the systemic, cellular, and subcellular levels (Ahrens et al., 2013; Antinucci and Hindges, 2016; Ahrens et al., 2013; Antinucci and Hindges, 2016). In models with induced obesity, for example, imaging tests can be used to measure the amount of fat mass gained from different diets, such as a high-fat diet (Landgraf et al., 2017; Landgraf et al., 2017). It can be performed on the same individual using different approaches, such as analyzing neural development. The use of two-photon microscopy enables visualization of biological structures and processes, allowing imaging of synapses, dendrites, and entire neuronal populations in *in vivo* assays with high resolution and complexity (Griffiths et al., 2020; de Vito et al., 2022; Griffiths et al., 2020; de Vito et al., 2022).

Two-photon excitation uses infrared light, promising less aggression and allowing greater tissue penetration without causing significant damage. It is invisible to fish, minimizing interference with natural behavior (Hasani et al., 2023). This methodology was applied to observe the process of adipogenesis in zebrafish, since the lipid metabolism pathways are the same in mammals (den Broeder et al., 2017). Thus, this method has also helped to investigate disease progression and synaptic changes in zebrafish models, similar to Alzheimer’s and Parkinson’s (Muto and Kawakami, 2016; de Vito et al., 2022). The application of the two-photon technique during pathological processes is ideal for observing morphological and functional changes, as well as the body’s response to potential treatments (Turrini et al., 2024). During the administration of peptide-based drug therapies, whose biodistribution still requires investigation, this technique allows direct visualization of peptide biodistribution and treatment responses *in vivo* (Turrini et al., 2023). Furthermore, as a translational animal model, using zebrafish, it is possible to observe whether a specific peptide can prevent synaptic degeneration or restore compromised neuronal function (Fiametti et al., 2025). The assessment of improvement in rapid axonal transport, a process that is also often dysfunctional in neurodegeneration, can also be monitored with two-photon after peptide administration (Grimaud et al., 2022).

Other methodologies have also been employed to investigate both disease mechanisms and treatment effects, including optogenetic analyses, which allow precise control of neuronal activity using light beams (Simmich et al., 2012). By expressing light-sensitive proteins, such as opsins, in specific neurons, it is possible to selectively activate or inhibit these neurons with light pulses, enabling the manipulation of neural circuits and the study of their functions (Zhu et al., 2009). Zebrafish represent an ideal model for optogenetics due to the ease of genetic manipulation, allowing the control of specific neuronal populations (Portugues et al., 2013; Chia et al., 2022). This approach provides insights into disease mechanisms and, when applied in therapeutic protocols, enables the evaluation of peptide efficacy in restoring circuit function or mitigating the effects of induced dysfunction (Zhang et al., 2024).

In addition to imaging approaches, biomolecular analysis methodologies, particularly gene expression profiling, allow for the understanding of the function and dysfunction of neural populations, complementing imaging by revealing the molecular mechanisms underlying disease and treatment effects (Denninger et al., 2022). Single-cell RNA sequencing (scRNA-seq) enables the analysis of gene expression profiles at the individual cellular level, uncovering cellular heterogeneity and transcriptional states that would be masked in bulk RNA analyses (Farnsworth et al., 2020). In zebrafish, scRNA-seq has been instrumental in mapping cellular development, identifying rare cell types, and understanding molecular changes that occur across different developmental stages and in response to stimuli (Pandey et al., 2023; Sur et al., 2023).

Studies in zebrafish have employed scRNA-seq to compare transcriptomic profiles in Alzheimer’s and Parkinson’s disease models with human tissues, revealing both shared and distinct molecular responses (Cosacak et al., 2022; Lau et al., 2025). This technique can identify early disease biomarkers, elucidate mechanisms of neuroinflammation and neurodegeneration, and guide the development of therapies targeting specific cell types (Cosacak et al., 2019). By analyzing the transcriptomic profiles of individual cells in neurodegenerative disease models treated with peptides, it is possible to identify the genetic pathways and cell types that respond to the intervention (Cosacak et al., 2019). This can reveal new therapeutic targets, treatment response biomarkers, and the heterogeneity of cellular responses to peptides, providing a detailed understanding of their molecular mechanisms of action (Cosacak et al., 2019).

Behavioral assays have been extensively used in zebrafish over the past decade to monitor performance alterations ranging from metabolic disorders to their neurodegenerative consequences (Godino-Gimeno et al., 2023). Recently, with advances in artificial intelligence (AI), its application has been further enhanced *in vivo* behavioral analyses, including the use of machine learning and computer vision, transforming the analysis of complex datasets generated in zebrafish studies (Fan et al., 2023). Additionally, AI algorithms are employed to process and interpret large volumes of imaging data, identifying subtle morphological and functional features that may be overlooked by conventional behavioral tests (Yang et al., 2021).

AI is particularly valuable for drug screening and disease model phenotyping, such as in Parkinson’s disease, where it has been employed to discriminate movement disorders, enabling the identification of novel therapeutic compounds (Gendelev et al., 2024). AI can also be used to assess the impact of genetic mutations or toxin exposures on behavior and neural morphology, providing a powerful tool for biomarker discovery and understanding disease pathogenesis (Bozhko et al., 2022; Del Rosario Hernández et al., 2024). In this context, AI can predict peptide efficacy based on their structure and interactions with biological targets, optimizing the drug discovery process and allowing a more precise evaluation of peptides’ ability to reverse or mitigate behavioral and morphological deficits associated with neurodegeneration (Del Rosario Hernández et al., 2024).

## 7 Therapeutic strategies assessed using zebrafish

Biomedical research heavily relies upon *in vivo* models for the evaluation and validation of new therapeutic strategies (Domínguez-Oliva et al., 2023). Such models, which include a variety of living organisms, allow the study of diseases in a complex biological context, providing crucial insights into the efficacy, safety and mechanisms of action of potential treatments (Lange and Inal, 2023). The application of animal models in biological studies and drug development has long been established, owing to the remarkable anatomical and physiological parallels between humans and mammals (Barré-Sinoussi and Montagutelli, 2015). The zebrafish has been deployed to investigate such effects, providing insights into how those peptides may influence brain function (Yan et al., 2024), simplified in Table 2.

**Table 2:** Therapeutic strategies evaluated in zebrafish models and their main effects, featuring different classes of molecules with neuroprotective and metabolic potential describing their mechanisms of action, observed effects, advantages, and limitation.

Molecule (therapeutic group)	Mechanism of action	Observed effects	Advantages	Limitations	References
Protein-based drugs					
Liraglutide (GLP-1 agonist receptor)	Activation of GLP-1 receptors; modulation of neuronal excitability and inflammation; improvement of glucose metabolism	Promoted neuronal viability, reduced apoptosis, regulated neuroinflammatory responses, and exhibited anxiolytic and antidepressant-like effects	Clinically approved; demonstrated neuroprotective efficacy in multiple models; translational potential to humans	Requires parenteral administration; possible desensitization of GLP-1R with chronic use	Candeias et al. (2015), Abd el-Rady et al. (2021), Bertelli et al. (2021), and Urkon et al. (2025)
Tirzepatide (GIP and GLP-1 receptor agonist)	Dual activation of GIP and GLP-1 receptors; AMPK activation; antioxidant, anti-inflammatory, and anti-apoptotic actions	Improved cognitive performance, restored antioxidant and anti-inflammatory markers in DM2 zebrafish models	Enhanced efficacy via dual incretin signaling; broader metabolic and neuroprotective coverage	Limited long-term data on CNS effects; potential gastrointestinal adverse events	Hristov et al. (2025) and Misra et al. (2025)
BDNF (neurotrophin)	Activation of TrkB receptors; modulation of synaptic plasticity and neuronal survival	Enhanced learning, memory, and synaptic connectivity; conserved function between zebrafish and humans	High translational relevance; potent modulator of synaptic function.	Poor pharmacokinetic stability; limited ability to cross the BBB	Cohen-Cory et al. (2010), De Felice et al. (2014), Lucini et al. (2018), and Lucon-Xiccato et al. (2022)
PACAP38 (neuropeptide)	Modulation of cAMP/PKA pathways; antioxidant, anti-apoptotic, and anti-inflammatory effects	Protected neurons and sensory cells from oxidative stress; mitigated apoptosis in neurotoxic models	Broad neuroprotective spectrum; highly conserved peptide	Short half-life; poor stability; complex receptor signaling network	Kasica et al. (2016), Kasica-Jarosz et al. (2018), Ghanizada et al. (2019), and Cherait et al. (2025)
NGF (neurotrophin)	Activation of TrkA receptors; regulation of neuronal differentiation, repair, and maintenance	Promoted neuronal survival, cholinergic protection, and synaptic repair	Central for neuronal development; strong neuroregenerative properties	Limited BBB permeability; potential pro-nociceptive and inflammatory effects	Bathina and Das (2015), Xiao and Le (2016), and Gu et al. (2025)
Non-protein drugs					
Metformin (biguanide)	AMPK pathway activation; regulation of mitochondrial respiration and insulin sensitivity	Exhibited neuroprotective effects, reduced oxidative stress, improved mitochondrial function and neuronal survival	Safe, inexpensive, and well-characterized; multi-targeted cellular benefits	Limited brain penetration; variable efficacy in advanced neurodegenerative stages	Sportelli et al. (2020) and Reed et al. (2025)
Vildagliptin (DPP-4 inhibitor)	Inhibition of dipeptidyl peptidase-4 enzyme; prolongation of endogenous GLP-1 activity	Enhanced neuronal survival, reduced oxidative damage, and improved behavioral parameters in DM2 models	Oral drug with good safety profile; indirectly promotes neurotrophic effects	Moderate CNS activity; limited experimental evidence in zebrafish	Pariyar et al. (2022)
*Sitagliptin* (DPP-4 inhibitor)	Inhibition of DPP-4, increasing levels of active incretins and insulin sensitivity	Reduced brain inflammation and oxidative stress; improved cognition in Parkinson-like zebrafish.	Neuroprotective potential through indirect incretin pathway activation	Mechanistic specificity in CNS remains unclear; moderate efficacy	Mani and Arfeen (2024)
Pioglitazone (thiazolidinediones)	PPAR-γ activation; regulation of mitochondrial and insulin signaling pathways; reduction of neuroinflammation	Improved cognitive and behavioral outcomes; decreased inflammatory cytokines and oxidative damage.	Well-known anti-inflammatory profile; promising in metabolic and neurodegenerative contexts	Associated with weight gain and cardiovascular side effects	Hu et al. (2024)
Rosiglitazone (thiazolidinediones)	PPAR-γ agonist; promotes mitochondrial biogenesis and glucose utilization in neurons	Enhanced learning performance; mitigated oxidative stress and synaptic dysfunction in zebrafish CNS models	Restores mitochondrial activity and synaptic plasticity	Safety concerns regarding cardiac effects; limited CNS selectivity	Cai et al. (2025)

These studies highlight the use of zebrafish as a translational model to investigate the relationship between metabolism, inflammation, and synaptic dysfunction.

Although zebrafish represent an attractive model for studies involving peptide-based therapeutic interventions, the route of administration and its influence on pharmacokinetics must be carefully considered when interpreting experimental outcomes. In larval models, immersion is the most commonly employed method; however, this approach relies on passive absorption through the skin and gills, which may not accurately reproduce systemic distribution and limits precise tracking of peptide uptake and biodistribution (Cocchiaro and Rawls, 2013). Moreover, peptide administration by immersion may increase susceptibility to degradation and instability, posing an additional challenge for therapeutic investigations (Lee et al., 2025).

In contrast, adult zebrafish allow more controlled delivery approaches, including intravenous and intraperitoneal microinjection as well as oral gavage, enabling better characterization of absorption, distribution, metabolism, and excretion (ADME) processes (Chaoul et al., 2023). In neurological studies, another important consideration is the developmental status of BBB. In larval zebrafish, BBB maturation is still ongoing, and the extent to which its permeability and functional properties resemble those of the mammalian BBB remains incompletely understood (Fleming et al., 2013). Consequently, BBB permeability may influence peptide exposure within the central nervous system and should be considered when extrapolating findings to mammalian models.

Furthermore, several studies have demonstrated that pharmacokinetic parameters obtained in zebrafish correlate with those observed in mammals, including humans. In particular, Kulkarni et al. highlighted the translational value of zebrafish for pharmacokinetic studies, supporting their use as a complementary model for predicting mammalian ADME profiles (Kulkarni et al., 2017). Therefore, differences in developmental stage and administration route should be taken into account when comparing peptide efficacy and pharmacokinetic profiles between zebrafish and mammalian models.

Due to its well-established central nervous system and ability to model a wide range of pathological conditions, from stress to DM2 and neurodegenerative diseases, zebrafish provide an efficient platform for screening and validating peptide compounds (Lucini et al., 2018). *In vivo* models have thus become crucial for elucidating metabolic dysfunction, such as that observed in DM2, accelerating neurodegenerative processes, including amyloid aggregation, mitochondrial impairment, and synaptic loss (Athauda and Foltynie, 2016; de Bem et al., 2021).

As previously mentioned, the association with metabolic disorders, such as DM2 and neurodegenerative diseases, has been increasingly recognized (Procaccini et al., 2016) and consequently, the number of studies focused on treating these diseases has also increased. Several studies have shown that certain classes of antidiabetic drugs can attenuate neuroinflammation, oxidative stress, mitochondrial dysfunction, and neuronal apoptosis, which are common pathological mechanisms in diseases such as Alzheimer’s and Parkinson’s (Nowell et al., 2023). The GLP-1, a derivative of proglucagon generated via post-translational processing, is primarily produced by enteroendocrine L cells in the intestine and can also be secreted by some neurons in the hindbrain, linking peripheral and central mechanisms of energy regulation (Nauck et al., 2021; Monti et al., 2022).

**Figure 4:**
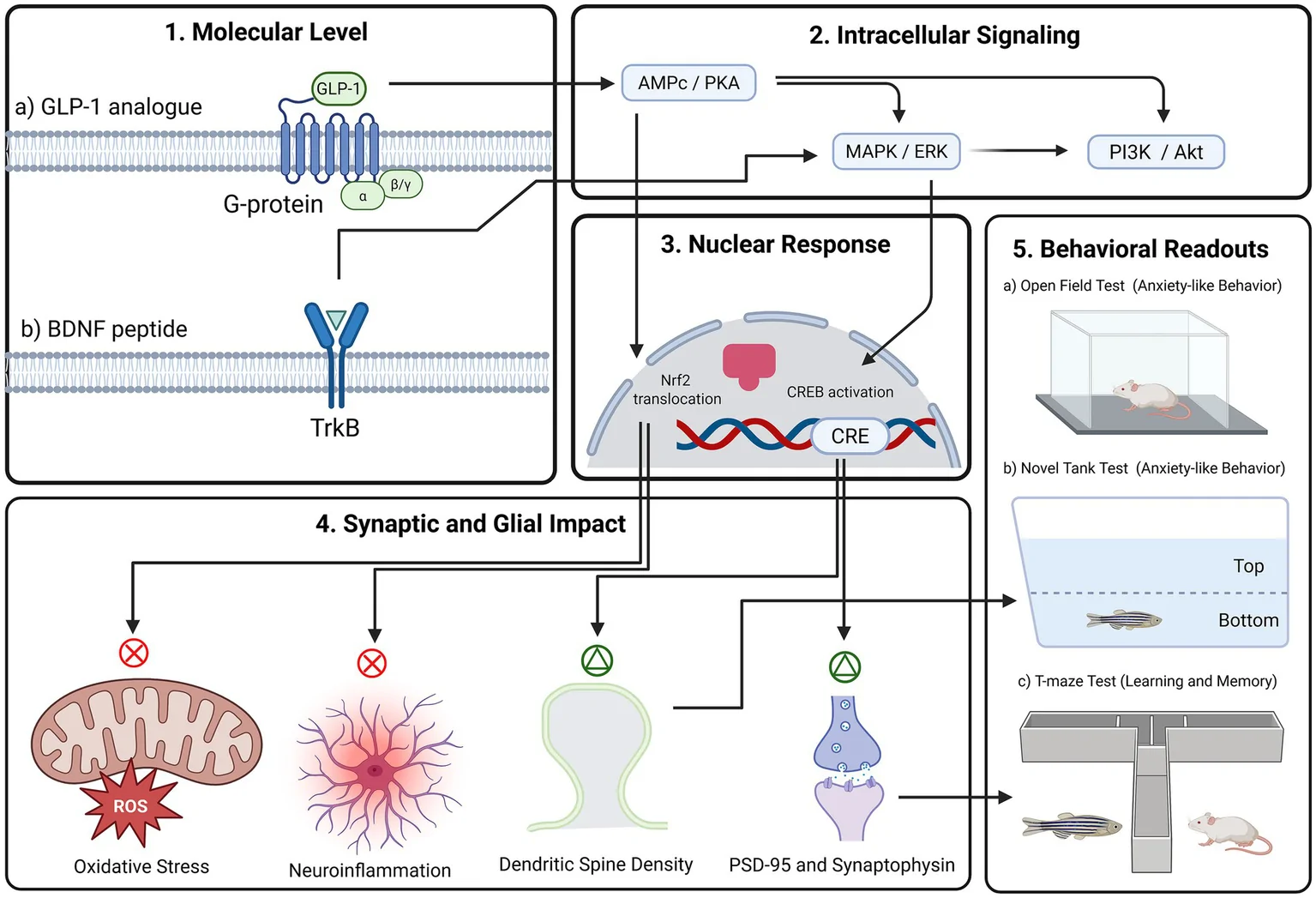
Proposed mechanisms of action of peptide-based therapeutics against obesity-associated synaptic dysfunction in zebrafish. GLP-1 analogs and BDNF activate their respective receptors (GLP-1R and TrkB), triggering intracellular signaling pathways (cAMP/PKA, MAPK/ERK, and PI3K/Akt) that promote Nrf2 and CREB activation. These pathways reduce oxidative stress and neuroinflammation while preserving dendritic spine density and synaptic proteins (PSD-95 and synaptophysin), ultimately improving behavioral outcomes. Red crossed circles indicate inhibited pathological processes, whereas green triangles represent preserved or enhanced neuroprotective effects. Figure created and modified by *BioRender.com* .

The GLP-1 and gastric inhibitory polypeptide (GIP) analogs, originally developed for the treatment of DM2, have shown promising neuroprotective effects, extending their therapeutic potential beyond glycemic regulation (Hristov et al., 2025). Moreover, Liraglutide, a GLP-1 receptor agonist, has been shown to promote neuronal viability and regulate neuroinflammatory processes in several *in vivo* models (Candeias et al., 2015; Urkon et al., 2025). Upon binding to GLP-1 receptors, Liraglutide activates intracellular signaling cascades, including the PI3K/Akt and MAPK/ERK pathways, which contribute to neuronal survival, attenuation of excitotoxicity, and enhancement of antioxidant defenses (Li et al., 2015). In particular, activation of these pathways has been associated with reduced oxidative stress and preservation of synaptic function. Notably, Bertelli et al. demonstrated that short-term liraglutide treatment exerts anxiolytic-like effects in zebrafish, whereas long-term administration mitigates the behavioral and physiological consequences of chronic unpredictable stress (Bertelli et al., 2021). Therefore, Liraglutide improves brain oxidative status and reduces oxidative damage, further supporting its neuroprotective properties (Bertelli et al., 2021; Lv et al., 2024), as can be seen below (Figure 4).

Beyond Liraglutide, another GLP-1 receptor agonist antidiabetic drug, Tirzepatide, a dual agonist of GIP and GLP-1 receptors, represents a potential neuroprotective drug (Hristov et al., 2025). Recently, a study in zebrafish with a high-fat diet induced by DM2 investigated the effects of Tirzepatide (10 nM/kg, i.p.), which improved cognitive performance and restored levels of GSH, catalase, and IL-10, effects attributed to its antioxidant, anti-inflammatory, anti-apoptotic, and AMPK-activating neuroprotective actions (Misra et al., 2025). The dual effects of Tirzepatide may provide a more robust and effective pharmacological profile compared to selective agonists, addressing the complex pathophysiology of diabetes-related cognitive impairment more comprehensively (Hristov et al., 2025; Misra et al., 2025).

Beyond hormonal peptides, such as Liraglutide and Tirzepatide, certain molecules may exert neuroprotective effects, including neurotrophic factors. Neurotrophic factors are proteins that regulate neuronal growth, differentiation, repair, and survival, playing a central role in synaptic transmission and neuronal plasticity (Mitre et al., 2016). Their effects are essential for neural integrity and plasticity, and the absence or dysfunction of these factors may lead to degeneration and apoptosis of neurons (Capossela et al., 2024). Synaptic modulation by those factors is crucial for the development and healthy functioning of the CNS (Durán Laforet and Schafer, 2024). *In vivo* studies have investigated the therapeutic potential of neurotrophic factors and synaptic modulators to restore neuronal function and connectivity in different neurological conditions (Numakawa and Kajihara, 2025).

BDNF is one of the most studied neurotrophic factors, known for its ability to modify synaptic strength and to act as a mediator, modulator, or instructor of synaptic plasticity (Cohen-Cory et al., 2010; Colucci-D’amato et al., 2020). Another important neuroprotective pathway involves brain-derived neurotrophic factor (BDNF) and its receptor TrkB. Activation of BDNF/TrkB signaling stimulates downstream PI3K/Akt and MAPK pathways (Teng et al., 2026), promoting neuronal survival, synaptic plasticity, and memory-related processes. Therefore, modulation of BDNF/TrkB signaling represents a promising strategy for counteracting obesity-associated synaptic dysfunction and cognitive impairment (Lee and Lim, 2025). Overall, these findings suggest that therapeutic peptides exert their beneficial effects through specific receptor-mediated signaling pathways that converge on antioxidant and pro-survival mechanisms, ultimately contributing to the preservation of synaptic plasticity and neuronal function.

Next, to neurotropic factors contributing to neuronal pathways, the pituitary adenylate cyclase-activating polypeptide (PACAP) is a neuropeptide with potential neuroprotective, anti-inflammatory, and antioxidant effects (Lucon-Xiccato et al., 2022). This neuropeptide is a highly conserved molecule, present mainly as PACAP38, its major isoform, and as the amidated isoform PACAP27 (Ghanizada et al., 2019). However, in zebrafish, only PACAP38 demonstrated protecting inner ear hair cells from oxidative stress, mitigating stress-induced apoptosis (Kasica et al., 2016). The distribution of peptides and their receptors in the zebrafish brain strongly suggests their involvement in cognitive and neuroprotective functions (Wu et al., 2006; Nakamachi et al., 2019).

**Figure 5:**
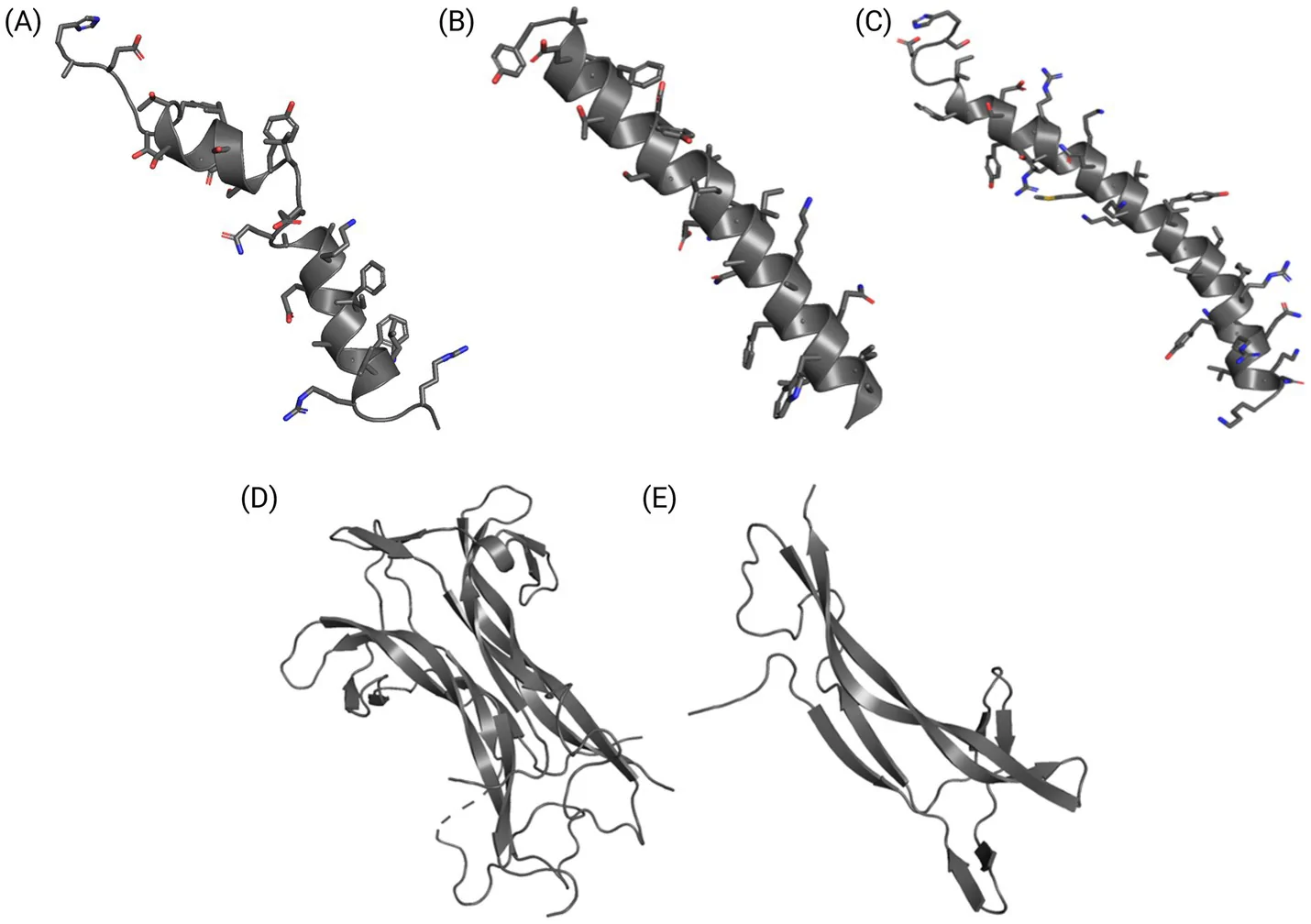
Three-dimensional molecular structures of protein drugs employed to treat metabolic disorders, using *Danio rerio* (Zebrafish) in vivo as a pharmaceutical model. **(A)** Liraglutide (4APD), **(B)** Tirzepatide (7FIM), **(C)** PACAP38 (2D2P), **(D)** BDNF (1B8M) and **(E)** NGF (6XUO). Figure visualized and modified using *PyMOL 3.1.8.*

The neuroprotective aspects of PACAP are attributed to its ability to modulate the inflammatory response, reduce oxidative stress, and promote cell survival, and in brain damage models, it can attenuate neuronal death while preserving neural function (Kasica-Jarosz et al., 2018; Cherait et al., 2025). Further neurotrophins, such as Nerve Growth Factor (NGF), are known due to their role in neuronal development and involvement in pain modulation and the inflammatory process (Bathina and Das, 2015). The NGF was the first neurotrophic factor discovered and is fundamental for the survival, differentiation, and maintenance of sensory and sympathetic neurons (Gu et al., 2025). NGF is abundant in the brain, especially in the hippocampus, contributing to neural development and helping to protect cholinergic neurons from degeneration (Xiao and Le, 2016). Therefore, developing humanized zebrafish models, where human genes are expressed in fish, promises to further refine the translational relevance of these studies (Moran et al., 2023). Additionally, understanding the three-dimensional molecular structures of drugs is fundamental to uncovering their mechanisms of action and optimizing therapeutic approaches, particularly when examining the complex interaction between obesity and metabolic and neurological dysfunctions (Lin et al., 2025). Protein-based therapeutics, particularly bioactive peptides, have gained increasing attention because they target conserved signaling pathways involved in obesity-associated synaptic dysfunction (Figure 5).

Among protein-based drugs, some display helical structure, namely Liraglutide, Tirzepatide, and PACAP38. These structural arrangements are often seen in hormone derived glucagon/secretin family, whose conformations adopt a helical structure when interacting with G protein-coupled receptors (Inooka et al., 2001). The prevalence of helixes within these drugs is closely associated with receptor-ligand binding affinity, whereby the C-terminal helix attaches to the extracellular domain of the receptor, allowing activation by the N-terminal (Runge et al., 2008). Meanwhile, BDNF and NGF belong to the neurotrophin family as opposed to helical peptides, which are characterized by their “cystine-knot” folding, featuring antiparallel *β*-sheets and dimeric structures (Robinson et al., 1999). These structural arrangements are typical for the neurotrophin family and allow for a specific binding surface for Trk receptors, responsible for activating survival and neuronal differentiation pathways (Robinson et al., 1999).

**Figure 6:**
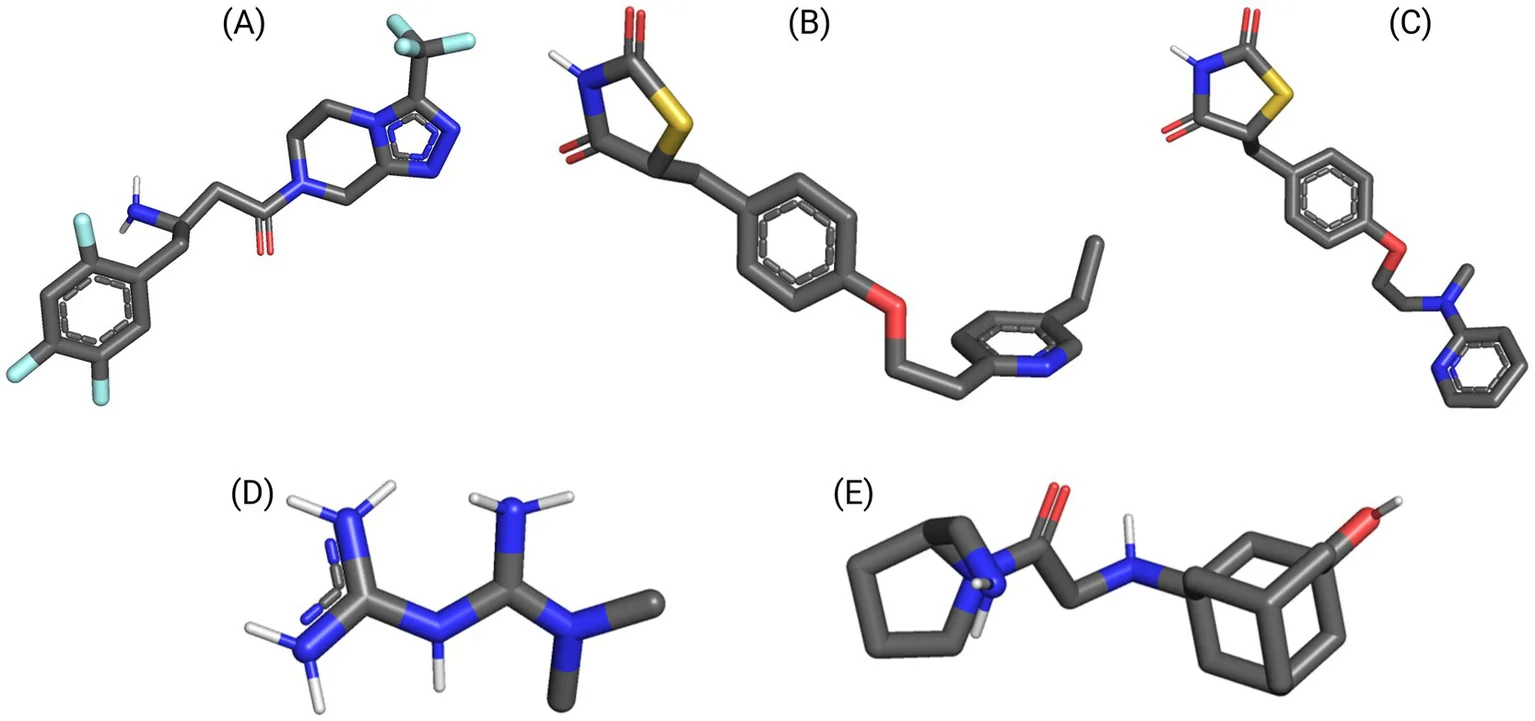
Three-dimensional molecular structure of non-protein drugs employed to treat metabolic disorders using *Danio rerio* (Zebrafish) in vivo as a pharmaceutical model. **(A)** Sitagliptin (PDB ID: 7Y4G), **(B)** Pioglitazone (PDB ID: 7 M26), **(C)** Rosiglitazone (PDB ID: 7 M24), **(D)** Metformin (PDB ID: 8Z7M) and **(E)** Vildagliptin (PDB ID: 6B1E). The molecular representations highlight the atoms by color (C: gray, H: white, N: blue, O: red, and S: yellow). Figure visualized and modified using *PyMOL 3.1.8.*

Apart from hormonal peptides, antidiabetic drugs also perform an essential part in regulating metabolic disorders. Metformin, a drug widely used for DM2, for example, has shown neuroprotective properties in animal models of neurodegenerative diseases, targeting various cellular pathways implicated in the progression of these conditions (Sportelli et al., 2020; Reed et al., 2025). Moreover, dipeptidyl peptidase-4 (DPP-4) inhibitors such as Vildagliptin and Sitagliptin have also exhibited neuroprotective effects in DM2 and Parkinson’s models, through mechanisms that are still being extensively investigated (Pariyar et al., 2022; Mani and Arfeen, 2024). Likewise, thiazolidinediones such as Pioglitazone and Rosiglitazone have demonstrated neuroprotective properties in animal models of diseases such as Parkinson’s and Alzheimer’s, resulting in improved behavioral performance and cognitive function (Hu et al., 2024). Furthermore, these findings highlight the potential for repositioning antidiabetic drugs for the treatment of neurological disorders, taking advantage of shared pathophysiological mechanisms such as insulin resistance and cerebral metabolic dysfunction (Cai et al., 2025). Non-protein drugs have also demonstrated promising therapeutic potential in zebrafish models of metabolic disorders (Figure 6).

These non-protein drugs present low-molecular-weight structures with different three-dimensional arrangements. Both Sitagliptin and Vildagliptin are DPP-4 inhibitors, an enzyme responsible for degrading incretins such as GLP-1 and GIP, hormones involved in regulating insulin secretion (Berger et al., 2018). Structurally, DPP-4 inhibitors present nitrogen heteroatoms, thereby constituting amide and nitrile groups, as well as hydrophobic aromatic rings (Lamos et al., 2019). The thiazolidinedione group, represented by Pioglitazone and Rosiglitazone, are characterized by the thiazolidinedione ring, which assists interaction with peroxisome proliferator-activated receptor gamma via hydrogen bonding, as well as a hydrophobic aromatic region that forms a cavity within the receptor, eventually, this process alters the gene expression related to lipid metabolism and insulin sensitivity (Ranjan et al., 2025). Metformin exhibits the simplest arrangement, a highly polar structure, as well as the presence of multiple amine groups (Rena et al., 2017). The simplicity of its structure is directly related to its mechanism of action, as it does not depend on specific binding to a particular receptor; thus, it acts primarily by activating AMP-activated protein kinase, leading to a reduction in hepatic glucose production and an increase in insulin sensitivity (Rena et al., 2017).

## 8 Translational limitations of zebrafish models

Despite the numerous advantages of the zebrafish model, there are limitations in usage, particularly regarding pharmacokinetics and peptide delivery (Kale et al., 2026). Although there is significant genomic and physiological similarity between zebrafish and humans, certain neuroanatomical differences may be a point of contention. For example, the zebrafish brain, particularly in the larval stage, lacks a laminated cerebral cortex, a structure essential for complex cognitive functions in mammals (Vohra et al., 2024). Furthermore, although zebrafish exhibit obesity induced by a high-fat diet, their short lifespan may differ in the temporal progression (Faillaci et al., 2018), meaning that scaling to a human lifetime is only approximate, not one-to-one. Thus, it is observed that, although the zebrafish serves as a noteworthy platform, research involving synaptic restoration and the efficacy of peptides must be validated through rigorous clinical trials to guarantee translational applicability.

Administration of therapeutic peptides using the water immersion method often results in unpredictable bioavailability, since absorption through the gills and skin differs substantially from mammalian routes of administration, thereby limiting the translational relevance of pharmacokinetic findings (Vyavahare et al., 2026). Consequently, researchers frequently employ parenteral routes, such as intraperitoneal, intravenous, or cerebroventricular administration, particularly in adult zebrafish when precise dosing and improved bioavailability are required. In contrast, larval zebrafish are generally exposed to compounds through water immersion, owing to their suitability for high-throughput screening. However, the use of parenteral administration in adults requires considerable technical expertise and reduces the efficiency of large-scale screening approaches (Kale et al., 2026; Vyavahare et al., 2026). Furthermore, the pharmacokinetics of mammalian peptides in zebrafish remain poorly characterized; differences in BBB permeability, rapid enzymatic degradation by piscine proteases (hydrolases), and altered clearance rates can significantly hinder the translation of effective therapeutic dosages from fish to mammalian systems (Fleming et al., 2013; Gupta et al., 2025). In fact, BBB permeability and the expression of drug transporters can vary among different *in vivo* model species, potentially leading to different pharmacokinetic responses (Liu et al., 2005).

About the pharmacokinetic constraints, notable divergences exist in metabolism, anatomical structures, and obesity phenotypes between zebrafish and mammals. While zebrafish possess conserved neuroendocrine pathways for appetite regulation, they lack encapsulated adipose depots equivalent to mammalian visceral and subcutaneous fat, storing lipids instead in pancreatic, dermal, and visceral regions without the same architectural complexity (Kulkarni et al., 2017; Zang et al., 2018). Brain anatomy also presents challenges; zebrafish lack a multi-layered neocortex, a region heavily implicated in the cognitive decline associated with metabolic syndrome in humans. This structural divergence complicates the direct translation of synaptic pathology, as the zebrafish telencephalon relies on different organizational principles to process spatial and emotional memory compared to the mammalian hippocampus and cortex (Picolo et al., 2021; Ghaddar and Diotel, 2022).

Finally, evaluating cognitive and behavioral deficits related to obesity-induced synaptic dysfunction in zebrafish carries inherent methodological limitations (Picolo et al., 2021). Although paradigms such as novel object recognition, Y-maze, and inhibitory avoidance tests are widely used to assess learning and memory, they are highly sensitive to confounding factors like altered locomotor activity or visual acuity, both of which can be perturbed by high-fat diets (Meguro et al., 2019; Picolo et al., 2021). Zebrafish behavior is heavily influenced by environmental stressors (e.g., water temperature, pH, and handling), which can increase inter-individual variability and obscure subtle cognitive rescues afforded by peptide interventions. Also, it has been observed that metabolic rates in ectothermic teleosts differ significantly from those of endothermic mammals, primarily because they do not possess brown adipose tissue (Zang et al., 2018), which may influence the presence of obesity-induced neuroinflammation. These constraints underscore the fact that while zebrafish are an exceptional screening tool for uncovering core molecular mechanisms, they must be viewed as a complementary model that requires subsequent validation in mammalian systems (Tao et al., 2022; Li et al., 2025).

## 9 Conclusion

This review establishes the zebrafish as a unique translational model, whose combination of neuro-metabolic conservation and advanced diagnostic technologies positions it as an ideal platform for elucidating the pathophysiology of obesity-induced synaptic dysfunction. Overall, it represents a balanced experimental system, being more cost-effective than mouse models while retaining greater biological complexity than *in vitro* approaches. As the field advances toward more complex peptide-based interventions, the zebrafish is increasingly integrated into the drug discovery pipeline, with growing acceptance in the pharmaceutical sector. Thus, its role is no longer a distant prospect but a consolidating reality, supporting the transition from molecular discovery to clinically relevant metabolic neurotherapies.
